# Senescent Thyrocytes, Similarly to Thyroid Tumor Cells, Elicit M2-like Macrophage Polarization In Vivo

**DOI:** 10.3390/biology10100985

**Published:** 2021-09-30

**Authors:** Mara Mazzoni, Giuseppe Mauro, Lucia Minoli, Loredana Cleris, Maria Chiara Anania, Tiziana Di Marco, Emanuela Minna, Sonia Pagliardini, Maria Grazia Rizzetti, Giacomo Manenti, Maria Grazia Borrello, Eugenio Scanziani, Angela Greco

**Affiliations:** 1 Molecular Mechanisms Unit, Department of Research, Fondazione IRCCS Istituto Nazionale dei Tumori, Via G.A. Amadeo, 42, 20133 Milan, Italy; mara.mazzoni@istitutotumori.mi.it (M.M.); giuseppe.mauro.m@gmail.com (G.M.); mcanania@libero.it (M.C.A.); tiziana.dimarco@istitutotumori.mi.it (T.D.M.); emanuela.minna@istitutotumori.mi.it (E.M.); sonia.pagliardini@istitutotumori.mi.it (S.P.); mariagrazia.rizzetti@istitutotumori.mi.it (M.G.R.); mariagrazia.borrello@istitutotumori.mi.it (M.G.B.); 2 Department of Veterinary Medicine, University of Milan, 26900 Lodi, Italy; lucia.minoli@unimi.it; 3 Mouse and Animal Pathology Laboratory (MAPLab), Fondazione Unimi, 20133 Milan, Italy; 4 Biomarker Unit, Department of Applied Research and Technical Development, Fondazione IRCCS Istituto Nazionale Tumori, 20133 Milan, Italy; loredana.cleris@istitutotumori.mi.it; 5 Animal Health and Welfare Unit, Department of Applied Research and Technical Development, Fondazione IRCCS Istituto Nazionale Tumori, 20133 Milan, Italy; giacomo.manenti@istitutotumori.mi.it

**Keywords:** thyroid carcinoma, senescence, thyrocytes, macrophages, inflammation, mouse xenografts

## Abstract

**Simple Summary:**

Several studies including ours suggest a pro-tumoral role of senescent thyrocytes in thyroid tumors. On the other hand, M2-like tumor associated macrophages infiltration increases with thyroid cancer aggressiveness. In this work, we used senescent thyrocytes and thyroid tumor cells as models of early and late tumor stages, respectively, and demonstrated their in vivo capability to recruit and polarize macrophages toward a pro-tumoral M2-like phenotype. These findings pave the way for the design of new therapeutic approaches for thyroid tumors based on the elimination or activity modulation of senescent cells and/or infiltrating macrophages.

**Abstract:**

Inflammation plays a critical role in thyroid cancer onset and progression. We previously characterized the in vitro interplay between macrophages and senescent human thyrocytes and thyroid tumor-derived cell lines, modeling the early and the late thyroid tumor phases, respectively. We reported that both models are able to induce pro-tumoral M2-like macrophage polarization, through the activation of the COX2-PGE2 axis. Here, we investigated the presence of macrophage infiltrating cells in mouse xenografts derived from the above described cells models. We showed that subcutaneous injection in immunodeficient mice of both senescent human thyrocytes and thyroid tumor-derived cell lines elicits macrophage recruitment. Furthermore, considering the type of macrophage infiltrate, we observed a stronger infiltration of Arginase I positive cells (M2-like). Overall, these results demonstrate the in vivo capability of senescent and tumor thyroid cells to recruit and polarize macrophages, suggesting that the promotion of a pro-tumoral microenvironment through tumor associated macrophages may occurs in late as well as in early thyroid tumor stages, favoring tumor onset and progression.

## 1. Introduction

Thyroid cancer is the most common endocrine malignancy and its incidence has increased in recent decades [1,2]. The majority of thyroid cancers arise from follicular cells and are generally classified as differentiated thyroid carcinomas (DTC) that include papillary thyroid carcinomas (PTC) and follicular thyroid carcinomas (FTC). DTC carry the best prognosis, usually being well treatable with surgery and radioactive iodine. Nevertheless, a fraction of DTC may progress into undifferentiated tumors. PTC accounts for about 80% of all thyroid cancers; it includes several histological variants differing in morphological pattern as well as in prognosis [3]. Among those, papillary thyroid microcarcinoma (PTMC) is the most indolent one; it is considered a low-risk cancer, and active surveillance has been proposed as a safe approach for a large set of patients [4,5]. Despite their generally indolent clinical course and good prognosis, a subset of PTMCs shows aggressive behavior [6]. Poorly differentiated and undifferentiated anaplastic thyroid carcinomas (PDTC and ATC, 5–10% and 2%, respectively), originate de novo or from pre-existing differentiated tumors. PDTC is characterized by intermediate aggressiveness between differentiated and undifferentiated cancers. ATC is extremely aggressive and displays poor prognosis; it represents a clinical challenge, since to date no effective therapies are available [7].

The association between cancer and inflammation is well known [8]. It is widely recognized that chronic inflammation represents a prerequisite to neoplastic transformation and tumor progression. The crucial role of the complex interplay between tumor cells and immune cells of the microenvironment in tumor development has emerged in virtually all cancers [8]. In this context, infiltrating macrophages are key players in many tumor types. Tumor associated macrophages (TAMs) support cancer cell survival, proliferation and invasion. This has led to many studies aimed at targeting TAMs by various approaches. TAMs appear more similar to M2 type polarized macrophages and are also designated M2-like [9].

The involvement of inflammation in thyroid cancer is well documented. In an in vitro setting, thyroid oncogenes are able to induce a proinflammatory program, including molecules able to recruit and educate macrophages, in primary thyrocytes [10,11]. TAMs are key players in thyroid carcinomas [12]. It has been reported that M2-like macrophage infiltration increases with thyroid cancer aggressiveness. The density of TAMs has been associated with aggressive features (presence of lymph node metastases and worse prognosis) in PTC, including also the generally indolent PTMC variant [12,13]. In PDTC, the extent of TAMs infiltration correlates with invasiveness and reduced survival [14], while in ATC, TAMs constitute a large part of the tumor mass in direct connection with cancer cells [15]. In addition, in a murine model of thyroid cancer, it has been demonstrated that targeting TAMs trough inhibition of CSF-1/CSF-1R impairs BRAFV600E-induced thyroid cancer progression [16].

A phenomenon closely related to inflammation is cell senescence. Oncogene-induced senescence (OIS) is triggered by oncogene expression in primary cells; it is characterized by growth arrest and considered a mechanism impeding transformation and tumor progression of oncogene-expressing cells and, more in general, inhibiting cancer development [17]. Indeed, senescent cells are often found in tumor early lesions [18]. OIS cells are metabolically active and produce a complex mixture of soluble factors, collectively indicated as SASP (sencence associated secretory phenotype), with multiple intrinsic and extrinsic effects, including a pro-tumoral effect by acting on tumor and microenvironment cells [19,20]. This highlights the dynamic nature of senescence process, and its dual and opposite effects on tumor evolution.

In thyroid cancer, we provided the first evidence of the occurrence of OIS, a finding later on confirmed by other authors. We demonstrated that senescence markers are upregulated in the generally indolent PTMC variant, and are progressively lost during thyroid tumor progression. We generated in vitro models of thyrocyte OIS, based on human primary thyrocytes transfected with PTC-associated oncogenes, thus modeling the early phases of thyroid cancer [21,22]. The pro-tumoral effect of senescent cells in thyroid cancer was proposed by Kim et al. [23]: they reported a frequent detection of senescent thyroid cells at the invasive front of BRAFV600E mutated human PTC and demonstrated that senescent cells promote tumor invasiveness. Very recently, we confirmed the presence of senescent cells at the PTC invasive front; we also documented the co-occurrence of cancer associated fibroblasts, thus proposing a possible co-operation of the two cell types in the invasive process [24].

The contribution of senescent thyrocytes to tumor progression is further corroborated by our recent studies in which we characterized the in vitro interplay between macrophages and senescent thyrocytes, modeling early thyroid tumor stages, in comparison with tumor-derived cell lines, modeling late stages. We have demonstrated that human monocytes exposed to conditioned medium from senescent thyrocytes and thyroid tumor cell lines undergo M2-like polarization, showing high CD206 and low MHC II markers, upregulation of CCL17 secretion, and tumor-promoting activity. We further reported that the prostaglandin-endoperoxide synthase enzyme (PTGS2/COX-2) gene, involved in the production of prostaglandin E2 (PGE2), is overexpressed in polarizing thyroid cells, demonstrating that the M2-like polarization ability is related to the production of PGE2 [25]. 

In this work, we characterize the interplay between macrophages and thyroid cells in in vivo settings, by means of senescent thyrocytes and thyroid tumor cell lines xenografts. The analysis of explants, showing a consistent amount of M2-like macrophages in both settings, demonstrates the in vivo capability of senescent and tumor thyroid cells to recruit and polarize macrophages and suggests that the promotion of a pro-tumoral microenvironment through tumor associated macrophages may occur in late as well as in early thyroid tumor stage, favoring tumor onset and progression.

## 2. Materials and Methods

### 2.1. Thyroid Cell Cultures

For the senescent cells, we used the ER:RAS inducible system previously established and characterized in our laboratory, based on primary human thyrocytes carrying an inducible H-RAS^G12V^ oncogene [22]. Upon tamoxifen (4OHT)-induced RAS expression, ER:RAS thyrocytes undergo senescence (senescent thyrocytes, STh), whereas untreated cells continue proliferating (proliferating thyrocytes, PTh). ER:RAS thyrocytes were cultured and treated as previously described [22,25]. The PTC-derived K1 cell line was grown in DMEM:Ham’sF12:MCDB (2:1:1). The other cell lines (PTC-derived NIM1; ATC-derived HTC/C3, FRO81–2, 8505C) were maintained in DMEM (Gibco, Life Technologies, Carlsbad, CA, USA) medium. All cell lines were cultured in medium supplemented with 10% (*v*/*v*) heat-inactivated fetal bovine serum (FBS, EuroClone, Pero, Italy) as monolayers at 37 °C in a 5% CO2 humidified atmosphere. Cell lines were authenticated by short tandem repeat (STR) profiles using the Stem Elite ID System (Promega, Madison, Wisconsin, United States) by the Fragment Analysis Facility at Fondazione IRCCS Istituto Nazionale dei Tumori. Cells were routinely tested for mycoplasma (PCR Mycoplasma Detection Set, TAKARA Bio Inc., Kusatsu, Japan).

### 2.2. In Vivo Studies

Female CD-1 nu/nu mice (5-weeks old) (Charles River, Calco, Italy) were injected subcutaneously with human senescent or proliferating thyrocytes (STh and PTh, respectively), or thyroid tumor cell lines. PTh or STh thyrocytes were mixed with Matrigel and injected subcutaneously in the flank; 5 × 10^5^ or 10^6^ cells were used; Matrigel alone was injected as control. In some cases animals were injected in both flanks; collectively 60 inoculums (22 Matrigel alone, 19 Sth, 19 Pth) in 36 animals were performed. Two different experimental setting were used: (1) inoculums of cells treated with 4OHT for 2 days, and explants after 1, 3, 4, 5 and 8 days; (2) inoculums of cells treated with 4OHT for 5 days, and explants after 1 and 2 days. The inoculated area, including the surrounding skin, subcutis and underlying skeletal muscle, was collected. Matched cell samples were followed in vitro and analyzed for the occurrence of senescence at time points corresponding to those of tissue explants. Thyroid tumor cell xenografts were produced in the context of another study. Thyroid tumor cell lines NIM1, K1 and FRO81-2 (5 × 10^6^ cells), 8505C (10 × 10^6^ cells), and HTC/C3 (20 × 10^6^ cells) were injected subcutaneously into the mice left flank. Tumor growth was assessed by monitoring tumor weight (TW) twice a week. TW was estimated by the formula TW(g) = d2 × D/2, where d and D are the shortest and the longest diameters, as previously described [26]. Mice were sacrificed 12–25 days after tumor cell injection, according to tumor growth rate. Tumors were explanted (TW from 0.2 to 0.9 g), including the surrounding skin, subcutis and underlying skeletal muscle. Animal studies were reviewed and approved by the Ethics Committee for Animal Experimentation of the hosting Institution and by Ministry of Health (Authorization numbers: 592/2017-PR; 761/2017-PR).

### 2.3. Histology and Immunohistochemistry

Explanted samples were fixed in 10% neutral-buffered formalin for 48 h and paraffin embedded. Four µm-thick sections from each sample were stained with Hematoxylin–Eosin (H&E) for histopathological evaluation. Additional serial sections were stained by immunohistochemistry with the following primary antibodies: anti-HLA (Abcam #ab52922; for the detection of cells of human origin); anti-Iba1 (Wako #019-19741; for the detection of macrophages); anti-iNOS (Abcam #Ab15323; for the detection of M1-like macrophages); anti-Arginase I (Santa Cruz Biotechnology #sc-18354; for the detection of M2-like macrophages). Heat-induced epitope retrieval was performed before incubating the slides with the primary antibody for one hour at room temperature. After incubation with the appropriate secondary biotinylated antibody, the slides were labelled by the avidin-biotin-peroxidase (ABC) procedure with a commercial immunoperoxidase kit (Vectastain Standard Elite, Vector Laboratories, Burlingame, CA, USA). The immunoreaction was developed with 3,3′-diaminobenzidine substrate (Vector Laboratories, Burlingame, CA, USA) for 5 min, and sections were counterstained with Mayer’s hematoxylin. For each marker, the amount of positive cells was semi-quantitatively scored. Scores has been defined as the percentage of the area occupied by positive cells (0 = absence; 0.5 = <5%; 1 = 5–10%; 2 = 10–25%; 3 = 25–50%). 

### 2.4. Western Blotting

Western blot analysis was performed as previously described [22]. The following antibodies have been used: p16INK4a (BD Biosciences, Milan, Italy); β-actin (Sigma-Aldrich, St Louis, MO, USA); anti-Pan-Ras (Calbiochem, San Diego, CA, USA); anti IL-8 (ab18672). Immunoreactive bands were visualized using horseradish peroxidase-conjugated secondary antibodies followed by enhanced chemiluminescence (GE Healthcare, Buckinghamshire, UK).

### 2.5. Proliferation Assay

Senescent and proliferating thyrocytes were plated in 96-well cell-culture plates. Proliferation was determined using the Cell Proliferation ELISA kit, BrdU (11669915001, Roche, Basel, Switzerland) following the manufacturer’s instructions.

### 2.6. Enzyme-Linked Immunosorbent Assay (ELISA) 

The levels of PGE2 in cell supernatants were measured by Prostaglandin E2 ELISA Kit Cat#: 514010 (Cayman Chemicals, MI, USA) according to the manufacturer’s instructions.

## 3. Results and Discussion

**Figure 1:**
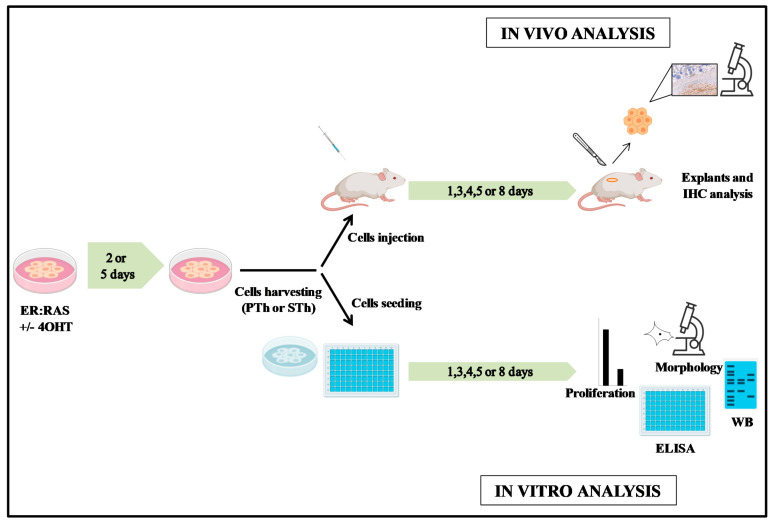
Flowchart of the senescent cells experimental design. 4OHT (Tamoxifen); Th (thyrocytes); PTh (proliferating thyrocytes); STh (senescent thyrocytes); IHC (immunohystochemistry).

We have previously reported that human monocytes exposed to conditioned medium from both senescent thyrocytes and thyroid tumor cell lines undergo M2-like polarization [25]. Here, we evaluated this issue in in vivo setting. We performed injection of senescent thyrocytes and thyroid tumor cell lines into nude mice. Although characterized by a limited immune system, this mouse model has been largely used to assess the macrophage infiltration in tumor xenografts of cell lines derived by different human tumor types. For the senescent cells we used the ER:RAS inducible system previously established and characterized in our laboratory [22]. It is based on primary human thyrocytes carrying an inducible H-RAS^G12V^ oncogene, in which senescence is achieved upon tamoxifen (4OHT)-induced RAS expression, whereas untreated cells continue proliferating. We excluded the possibility to induce senescence of ER:RAS thyrocytes by treating injected mice with 4OHT, as it has been reported that 4OHT treatment in mice significantly decreases the expression of molecules involved in monocyte chemotaxis [27]. We exploited subcutaneous injection of in vitro induced senescent thyrocytes. Specifically, senescent or proliferating thyrocytes (STh and PTh, respectively) or Matrigel alone were injected in immunodeficient mice, as reported in Materials and Methods section. The experiments hereafter described were performed according to the flowchart in Figure 1. 

**Figure 2:**
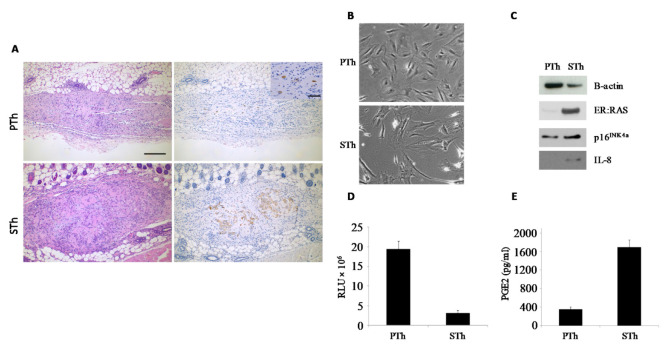
Analysis of PTh and STh explants and in vitro matched samples. ER:RAS thyrocytes were treated with 4OHT in vitro for 2 days and then collected for the injection in mice; matched cell samples were followed in vitro and analyzed at the moment of the explants (3 days post injection, 5 days from senescence induction). (**A**) Explants’ sections were stained with H&E (left) and HLA (right). Rare HLA-positive human thyrocytes present in PTh group are shown in the inset. 100× magnification, BAR = 200 μm; inset: 400× magnification, BAR = 50 μm. (**B**–**E**) Analysis of in vitro cell matched samples for the presence of senescence markers. (**B**) Representative pictures showing cell morphology (10× magnification). (**C**) Western blot analysis for the expression of ER:RAS, p16INK4a and IL-8 proteins (β-actin represents loading control). (**D**) BrdU incorporation assay for cell proliferation. (**E**) Determination of PGE2 secretion, by ELISA. RLU: relative luminescence unit.

As a first approach, we injected mice with proliferating thyrocytes (PTh) or thyrocytes undergoing senescence (STh) upon in vitro 4OHT-treatment for 2 days; in this setting, senescence features are evident as early as day 3 (Figure S1) and last at least 14 days [25]. Animals were sacrificed at day 1, 3, 4, 5 and 8 post injection, and explants were analyzed for the presence of cells of human origin by means of anti-HLA immunohistochemistry. In H&E sections, the subcutis was focally expanded by a nodular deposit of pale eosinophilic amorphous material (consistent with Matrigel) associated with a moderate cellular infiltrate characterized by macrophages, sparse neutrophils and variable number of larger cells with oval nuclei, vesicular chromatin and single or multiple evident nucleoli, consistent with injected thyrocytes. Indeed, HLA immunohistochemistry confirmed their human origin. The comparison between samples explanted at different time points, showed that the number of human thyrocytes decreased with time in both PTh and STh experimental settings, and in some samples no HLA-positive cells were detected. Of note, the cell number decrease was more evident for PTh than STh, suggesting that senescent cells survive longer than proliferating ones, likely because of their ability to produce survival factors. A representative example is shown in Figure 2A.

Cell samples matched with the injected ones were followed in vitro, and analyzed at time points corresponding to those of tissue explants. The occurrence of senescence was confirmed by assessing: morphology, expression of ER:RAS, p16INK4a and IL-8 proteins (by WB), cell proliferation (by BrdU incorporation assay), and level of secreted PGE2 (by ELISA) (Figure 2B–E and Figure S2). 

**Figure 3:**
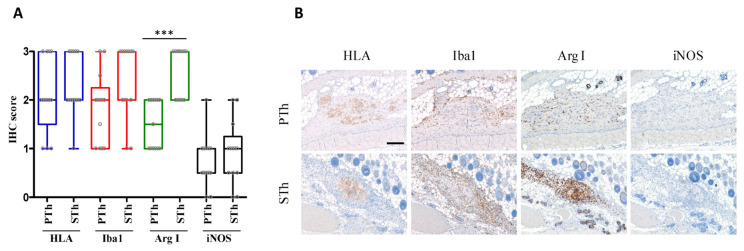
Immunohistochemical analysis of PTh and STh explants. (**A**) Boxplot with scatterplot showing IHC score relative to the markers HLA, Iba1, ArgI and iNOS evaluated in proliferating thyrocytes (PTh) and senescent thyrocytes (STh) explants. Scores has been defined as the percentage of positive areas (0 = absence; 0.5 = <5%; 1 = 5–10%; 2 = 10–25%; 3 = 25–50%). *** *p*-value < 0.0001. Samples from both experimental approaches (see Materials and Methods), displaying a comparable amount of HLA-positive cells were considered (PTh *n* = 13; STh *n* = 13). (**B**) Explants analysis performed at 1 day post-injection of PTh and STh (5 days 4OHT-treated) cells. HLA, Iba1, Arginase I, iNOS immunohistochemistry. 100× magnification, BAR = 200 μm.

We then applied a second approach based on injection of senescent thyrocytes (obtained after 5 days of in vitro 4OHT treatment), and mice were sacrificed after 1 and 2 days. In this experimental setting, the number of HLA-positive cells identified in the explants, scattered distributed within Matrigel deposits, was similar for both groups. Xenograft samples of PTh and STh from both experimental approaches, displaying a comparable amount of cells of human origin (HLA-positive), were analyzed for macrophage infiltrate by Iba1 immunostaining. The injection of human thyrocytes elicited a more intense macrophage reaction than the injection of Matrigel alone; the data are reported in Tables S1–S3. PTh and STh explants were also analyzed for macrophage polarization by immunostaining for M1 and M2 markers (iNOS and Arginase I). No significant differences between PTh and STh explants were observed with respect to the number of Iba1-positive cells. However, the immunostaining for M1 and M2 macrophage markers indicated a higher amount of M2-like macrophages (Arginase I-positive cells) in STh explants compared with PTh group, while no significant differences were observed for M1-like macrophages (iNOS-positive cells), which were less frequently detected in both groups (Figure 3, Tables S2 and S3). Unfortunately, the limited amount of explanted material did not allow for additional analyses. 

Our results suggest that, in an in vivo setting, STh are able to recruit macrophages at the site of injection, and to trigger an M2-like polarization. Other authors reported the ability of senescent cells to influence macrophages in vivo. Xu M. and collaborators demonstrated that senescent fibroblasts transplantation in knee joint region of mice promotes an osteoarthritis-like condition, characterized by inflammation and immune cells infiltration; interestingly, transplanted senescent cells are able to recruit macrophages by the secretion of the macrophage chemoattractant MCP-1 [28]. Moreover, it has been reported that senescent cells can affect macrophages polarization [29]. Our study suggests that senescent thyrocytes (OIS) present in PTMC may be capable to establish a pro-tumoral microenvironment by triggering M2-like macrophage polarization; this could potentially represent one of the mechanisms leading to the eventual progression and poor outcome of PTMC in a subset of patients. In this scenario, it is intriguing to speculate that targeting senescent cells within a PTMC tumor mass would contribute to maintain the indolent course of PTMC. Further studies investigating the extent of senescence as a prognostic factor for PTMC are needed.

**Figure 4:**
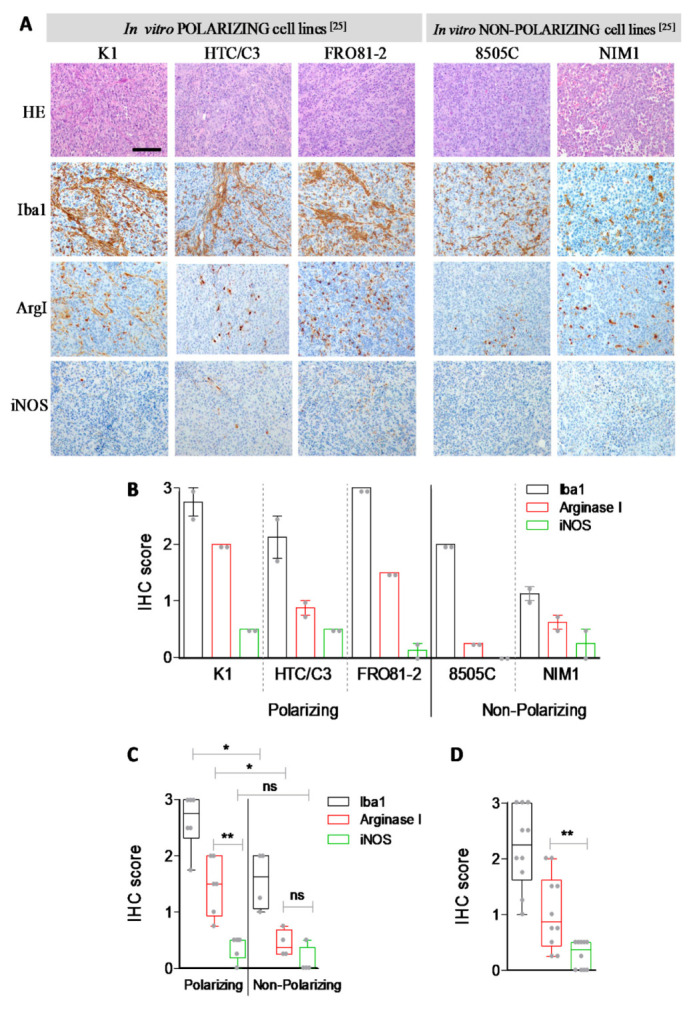
Immunohistochemical analysis of tumor explants. (**A**) H&E, Iba1, ArgI and iNOS immunohistochemistry in thyroid tumor xenografts; representative pictures of the indicated tumor cell lines tumor explants. 200× magnification, BAR = 100 μm (**B**) Barplot (mean ± SEM) with scatterplot showing IHC scores of the corresponding cell lines (see material and methods); two animals for each cell line were tested. The same results are showed (**C**) in samples stratified for in vitro M2-like polarization capacity or (**D**) as unique class. Each dot represents IHC score computed as mean of at least four (40×) fields scored for each xenograft (see Figure S3). Statistical significance by non-parametric Mann–Whitney test; ** *p*-value < 0.01 and * *p*-value < 0.05.

We have previously reported that, similarly to senescent cells, conditioned medium from thyroid tumor cell lines, modeling thyroid tumor late stage, is able to induce in vitro M2-like polarization of human monocytes. To investigate this issue in an in vivo setting, thyroid tumor cells xenografts were analyzed for the presence of macrophages. Specifically, we analyzed thyroid tumor explants from cell lines previously scored positive (K1, HTC/C3 and FRO81–2) or negative (8505C and NIM1) for the capacity of inducing in vitro M2-like polarization [25]. Evaluation of macrophage infiltrates (Iba1) and macrophage polarization status (iNOS and Arginase 1) was performed. Macrophage infiltration was detected in all the xenografts; representative IHC results for each cell line are shown in Figure 4A and Figure S3. The quantification of IHC scores deriving from the analysis of two different xenografts for each cell line is shown in Figure 4B. The distribution of IHC scores shows an overall higher number of Iba1-positive cells and a higher proportion of Arginase 1-positive cells in xenografts from in vitro polarizing K1, HTC/C3 and FRO81–2 cell lines, compared to those from non-polarizing cell lines 8505C and NIM1 (Figure 4C). Interestingly, as shown in Figure 4 and in Figure S3, M2-like macrophages, although at significantly lower numbers, were detected also in 8505C and NIM1 cell lines previously scored non-polarizing by in vitro assay [25]. This is in agreement with other reports showing the presence of M2-like macrophages infiltration in tumor xenografts derived from different thyroid tumor cell lines, including 8505C [30]. The discrepancy with our previous study [25] may simply reflect the general limitations of in vitro analyses, which, most likely, underestimated cell lines polarization capabilities. Moreover, thyroid tumor cells xenografts present a consistent infiltration of Iba-1/Arg1-positive M2-like polarized macrophages (Figure 4D), thus corroborating our previous in vitro results. 

## 4. Conclusions

Overall, our results demonstrate the in vivo capability of senescent and tumor thyroid cells to recruit and polarize macrophages toward an M2-like phenotype, thus promoting a pro-tumoral microenvironment. Therefore, it is intriguing to envisage therapeutic approaches for thyroid tumors based on the elimination or activity modulation of senescent cells and/or infiltrating macrophages.

## Data Availability

Not applicable.

## Supplementary Materials

The following are available online at https://www.mdpi.com/article/10.3390/biology10100985/s1, Figure S1: PTh and STh in vitro analysis, Figure S2: Densitometric analysis, Figure S3: Iba1, Arg1 and iNOS immunoistochemistry in thyroid tumor xenografts, Table S1: Iba1 IHC score in Matrigel samples, Table S2: PTh IHC scores, Table S3: STh samples IHC scores.
