# Supplementary material for: Senescent Thyrocytes, Similarly to Thyroid Tumor Cells, Elicit M2-like Macrophage Polarization In Vivo
Source: Biology (Basel). 2021 Sep 30;10(10):985. doi: 10.3390/biology10100985 (PMC8533427; doi:10.3390/biology10100985)
Supplement: Supplementary file 1 [file biology-10-00985-s001.zip › supplementary/biology-1340174-WB.pdf]

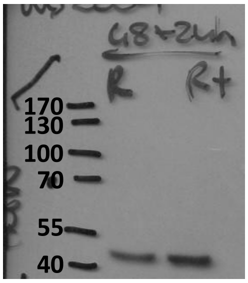


**B-actin**

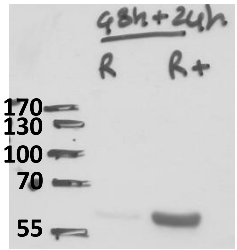


**Pan-RAS**

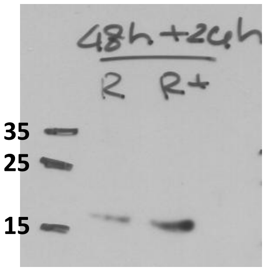


**p16**

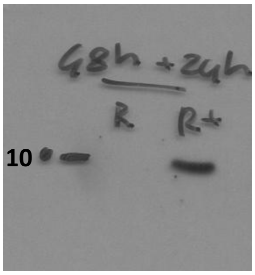


**IL-8**

**Original film corresponding to the WB panel shown in Figure S1**. Blot was immunoblotted with the indicated antibodies. "R" stands for "PTh" (untreated ER:RAS thyrocytes); "R+" stands for "STh" (4OHT-treated ER:RAS thyrocytes). "48h+24h" stands for "48 hours of *in vitro* coltures + 24 hours (time of the explants, from the moment of injection, for the correspondent *in vivo* injected cells). Molecular weight markers are indicated (kDa)

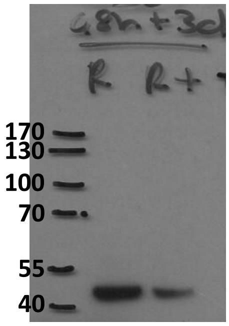


**B-actin**

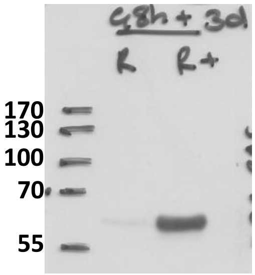


**Pan-RAS**

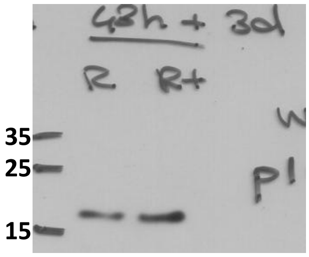


**p16**

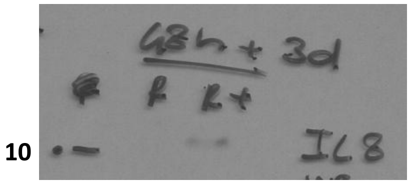


**IL-8**

**Original film corresponding to the WB panel shown in Figure 2C**. Blot was immunoblotted with the indicated antibodies. “R” stands for “PTh” (untreated ER:RAS thyrocytes); “R+” stands for “STh” (4OHT-treated ER:RAS thyrocytes). “48h+3d” stands for “48 hours of *in vitro* coltures + 3 days (time of the explants, from the moment of injection, for the correspondent *in vivo* injected cells). Molecular weight markers are indicated (kDa)
