# Supplementary material for: Senescent Thyrocytes, Similarly to Thyroid Tumor Cells, Elicit M2-like Macrophage Polarization In Vivo
Source: Biology (Basel). 2021 Sep 30;10(10):985. doi: 10.3390/biology10100985 (PMC8533427; doi:10.3390/biology10100985)
Supplement: Supplementary file 1 [file biology-10-00985-s001.zip › supplementary/Figure S1.pdf]

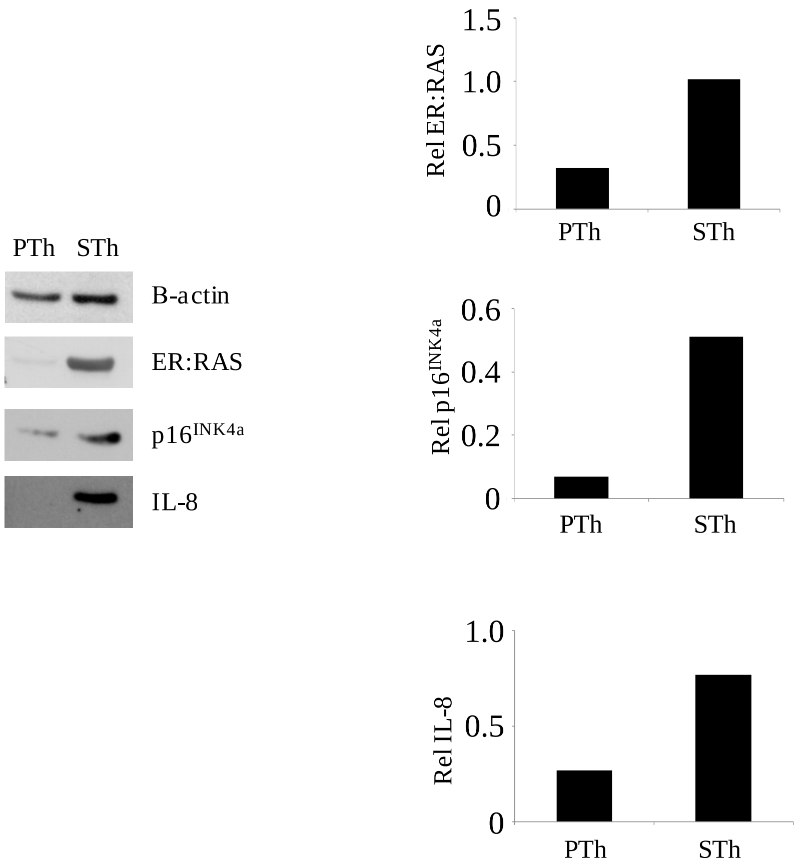

Figure S1. PTh and STh in vitro analysis. ER:RAS thyrocytes were treated with 4OHT, in vitro, for 2 days and then collected for the injection in mice; matched cell samples were followed in vitro and analyzed at the moment of the explants (1 day post injection, 3 days from senescence induction). (**left panel**) Western blot analysis for the expression of ER:RAS, p16INK4a and IL-8 proteins (β-actin represents loading control). (**right panel**) Densitometric analysis of ER:RAS, p16INK4a and IL-8 protein levels expressed as ratio on β-actin level
