# Supplementary material for: Senescent Thyrocytes, Similarly to Thyroid Tumor Cells, Elicit M2-like Macrophage Polarization In Vivo
Source: Biology (Basel). 2021 Sep 30;10(10):985. doi: 10.3390/biology10100985 (PMC8533427; doi:10.3390/biology10100985)
Supplement: Supplementary file 1 [file biology-10-00985-s001.zip › supplementary/Figure S2.pdf]

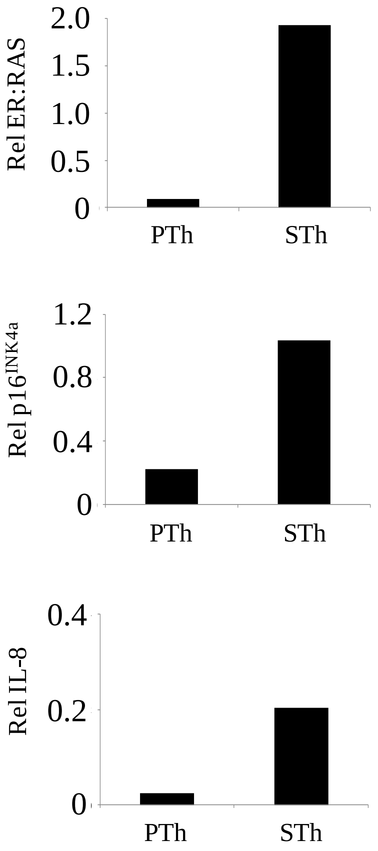

Figure S2. Densitometric analysis. ER:RAS, p16INK4a and IL-8 protein levels expressed as ratio on β-actin level (referred to Figure 2C).
