# Supplementary material for: Senescent Thyrocytes, Similarly to Thyroid Tumor Cells, Elicit M2-like Macrophage Polarization In Vivo
Source: Biology (Basel). 2021 Sep 30;10(10):985. doi: 10.3390/biology10100985 (PMC8533427; doi:10.3390/biology10100985)
Supplement: Supplementary file 1 [file biology-10-00985-s001.zip › supplementary/Figure S3.pdf]

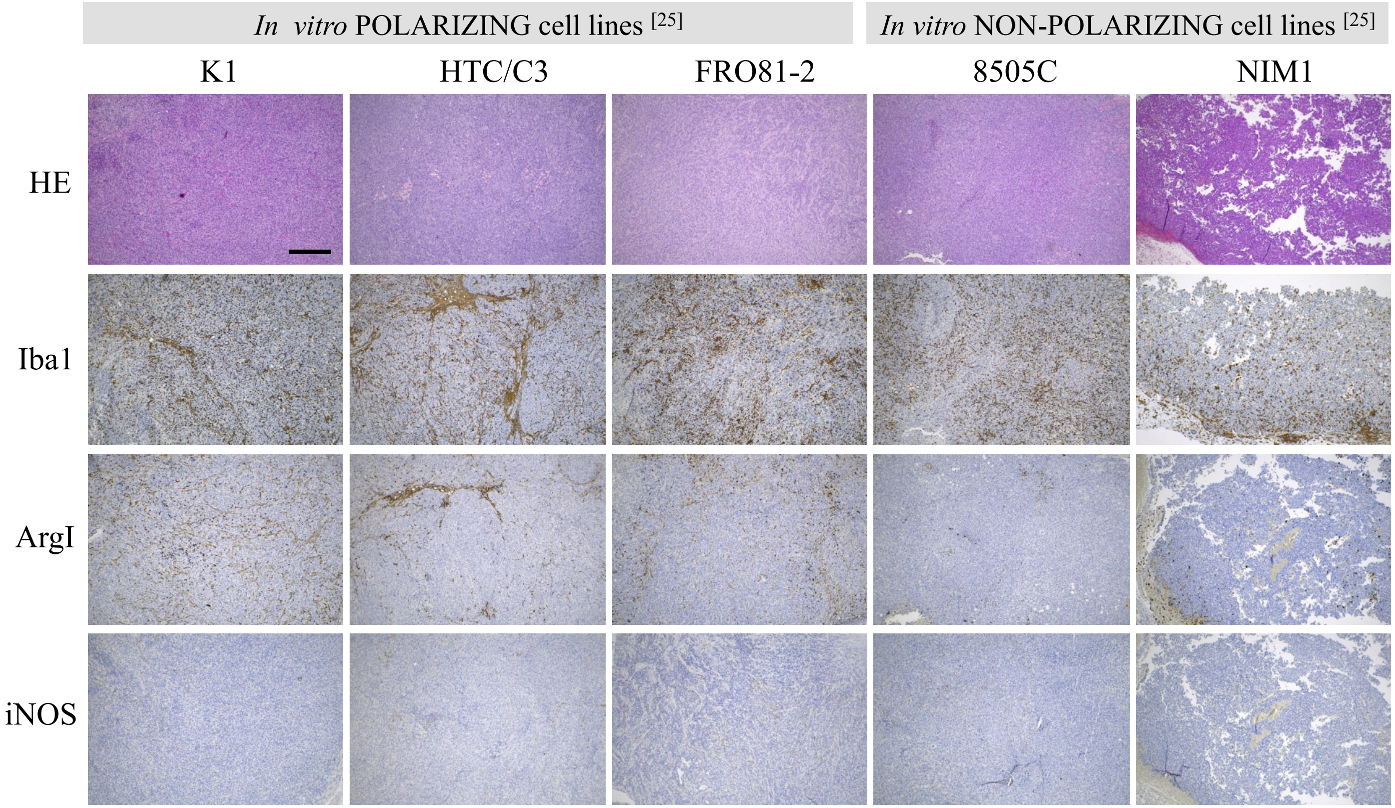


**Figure S3. Immunohistochemical analysis of tumor explants.** H&E, Iba1, ArgI and iNOS immunohistochemistry in thyroid tumor xenografts; representative pictures of the indicated tumor cell lines tumor explants. 40x magnification, BAR= 500µm
