# Supplementary material for: Senescent Thyrocytes, Similarly to Thyroid Tumor Cells, Elicit M2-like Macrophage Polarization In Vivo
Source: Biology (Basel). 2021 Sep 30;10(10):985. doi: 10.3390/biology10100985 (PMC8533427; doi:10.3390/biology10100985)
Supplement: Supplementary file 1 [file biology-10-00985-s001.zip › supplementary/Table S1.pdf]

	M Explants	
	Explant (days)	Iba1 score
**M #1**	1	0.5
**M #2**	1	n.e.
**M #3**	1	0.5
**M #4**	2	0.5
**M #5**	2	0.5
**M #6**	3	0.5
**M #7**	3	0.5
**M #8**	3	0.5
**M #9**	3	0.5
**M #10**	5	0.5
**M #11**	5	0.5
**M #12**	5	0.5
**M #13**	5	0.5

Table S1. M IHC scores. IHC score relative to the marker Iba1, evaluated in matrigel only explants (M). For each sample, scores derived from the average of two microscopic fields; scores has been defined as the percentage of the matrigel area occupied by positive cells (0= absence; 0.5= <5%; 1= 5-10%; 2= 10-25%; 3= 25-50%). For each sample is indicated the time of explant.
