# Supplementary material for: Senescent Thyrocytes, Similarly to Thyroid Tumor Cells, Elicit M2-like Macrophage Polarization In Vivo
Source: Biology (Basel). 2021 Sep 30;10(10):985. doi: 10.3390/biology10100985 (PMC8533427; doi:10.3390/biology10100985)
Supplement: Supplementary file 1 [file biology-10-00985-s001.zip › supplementary/Table S2.pdf]

	PTh Explants				
	Explant (days)	HLA score	Iba1 score	ArgI score	iNOS score
**PTh #1**	1	3	1	1	0.5
**PTh #2**	1	2	2	1	0.5
**PTh #3**	1	2	2	2	1
**PTh #4**	1	2	1	1.5	0.5
**PTh #5**	2	1	2	2	0.5
**PTh #6**	2	2	1	2	0
**PTh #7**	2	3	1	1	1
**PTh #8**	2	1	2	1	1
**PTh #9**	2	2	2	2	2
**PTh #10**	1	3	1.5	1	1
**PTh #11**	3	1	3	1	0.5
**PTh #12**	4	3	3	2	0
**PTh #13**	5	2	2.5	2	1

Table S2. PTh samples IHC scores. IHC score relative to the markers HLA, Iba1, ArgI and iNOS evaluated in proliferating thyrocytes (PTh) explants. For each sample, scores derived from the average of two microscopic fields; scores has been defined as the percentage of the matrigel area occupied by positive cells (0= absence; 0.5= <5%; 1= 5-10%; 2= 10-25%; 3= 25-50%). For each sample is indicated the time of explant.
