# Supplementary material for: Senescent Thyrocytes, Similarly to Thyroid Tumor Cells, Elicit M2-like Macrophage Polarization In Vivo
Source: Biology (Basel). 2021 Sep 30;10(10):985. doi: 10.3390/biology10100985 (PMC8533427; doi:10.3390/biology10100985)
Supplement: Supplementary file 1 [file biology-10-00985-s001.zip › supplementary/Table S3.pdf]

	STh Explants					
	**4OHT *in vitro* induction (days)**	**Explant (days)**	**HLA score**	**Iba1 score**	**ArgI score**	**iNOS score**
**Sth #1**	5	1	3	2	3	1
**Sth #2**	5	1	2	1	2	0.5
**Sth #3**	5	1	2	2	3	1
**Sth #4**	5	1	2	2	2	1
**Sth #5**	5	2	3	2	3	1
**Sth #6**	5	2	3	1	3	0.5
**Sth #7**	5	2	2	3	3	2
**Sth #8**	5	2	2	3	3	2
**Sth #9**	5	2	2	3	3	1
**Sth #10**	2	1	3	3	2	0.5
**Sth #11**	2	3	3	3	2	1.5
**Sth #12**	2	4	1	3	3	0
**Sth #13**	2	5	2	3	2	0

Table S3. STh samples IHC scores. IHC score relative to the markers HLA, Iba1, ArgI and iNOS evaluated in senescent thyrocytes (STh) explants. For each sample, scores derived from the average of two microscopic fields; scores has been defined as the percentage of the matrigel area occupied by positive cells (0= absence; 0.5= <5%; 1= 5-10%; 2= 10-25%; 3= 25-50%). For each sample are indicated the time of treatment with 4OHT *in vitro*, and the time of explant.
